# Prevalence and factors associated with post traumatic stress disorder among field police patrol officers serving in Kampala Metropolitan region

**DOI:** 10.1186/s12888-022-04317-z

**Published:** 2022-11-15

**Authors:** Rogers Agenda Isabirye, Justine Diana Namuli, Eugene Kinyanda

**Affiliations:** 1 Department of Psychiatry, Makerere University, College of Health Sciences, Kampala, Uganda; 2 Mental Health Section, MRC/UVRI & LSHTM Uganda Research Unit, Entebbe, Uganda

**Keywords:** Trauma, Police, Field work, Africa, CAPS

## Abstract

**Background:**

Occupation groups like police officers and fire fighters are exposed to a number of traumatic events which put them at a risk of developing post-traumatic stress disorder (PTSD). Previous studies have found the prevalence of PTSD in police officers to vary between 7 and 19%. However, most of these studies have been undertaken in western setting with little research having been undertaken in sub-Saharan Africa including Uganda.

**Objective:**

To determine the prevalence and factors associated with post-traumatic stress disorder among field police patrol officers serving in Kampala Metropolitan Police (KMP) North Region.

**Methods:**

This was a cross sectional study that was conducted on 392 field police patrol officers serving in KMP North Region. Diagnosis of PTSD was undertaken using the Clinician Administered PTSD Scale for DSM-5. In order to assess for psychiatric comorbidities, the study used the Mini International Neuropsychiatric Interview (M.I.N.I.)

**Results:**

In this study, the prevalence of PTSD was 7.4%. An additional 62.5% had sub-threshold PTSD, which was defined as, the presence of at least one PTSD symptom but not meeting full criteria for PTSD diagnosis. The factors found to be significantly associated with PTSD were all related to the presence of psychiatric comorbidities, namely the presence of: a current major depressive episode (aOR = 4.7; 95% CI: 1.5- 14.8; *p* = .009); an alcohol use disorder (aOR = 5.1; 95% CI: 2.0–13.0; *p* = .001); and presence of dissociation symptoms (aOR = 6.7; 95% CI: 2.0–22.2; *p* = .002).

**Conclusion:**

PTSD is one of the common psychiatric disorders experienced by serving police officers in Uganda. The tendency of PTSD in this group to co-occur with other psychiatric disorders means that any treatment program to address it should be part of a comprehensive multi-disorder mental health treatment programme in the police office.

## Background

Post-traumatic stress disorder (PTSD) is a psychiatric disorder which follows exposure to a traumatic event [1]. Exposure to traumatic events has been found to be higher in some occupations like the police where they may experience at least three traumatic events for every six months of service [17]. In addition to being in situations in which they are at risk of being injured or killed, police officers are often exposed to persons who have been injured or killed because of road traffic accidents, murders, suicides, and other incidents [10]. Police officers who handle dead bodies can also be traumatised by visual, tactile, and olfactory sensations [10]. One study noted that officers report feelings of powerlessness and despair following incidents involving injury or death to children [16]. The recent outbreak of COVID-19 pandemic has forced a number of countries to enforce lock down, social distancing and curfews. Security personnel like police officers have been tasked to enforce these rules and have had to attend to the exponential rise in domestic violence related crimes at the risk of acquiring COVID-19 and developing mental health issues like PTSD and anxiety disorders [40, 51].

There are a number of negative health consequences that can occur among police officers due to exposure to traumatic events. These include; poorer sleep quality [30], and a high prevalence of some mental health problems like alcohol abuse and dependence [31], PTSD and depression [34, 43]. PTSD symptoms have been significantly associated with increased general health symptoms and conditions, poorer physical health-related quality of life, greater frequency and severity of pain, and cardio-respiratory and gastrointestinal complaints [33].

PTSD presents with a number of symptoms such as re-experiencing the traumatic event, avoidance of situations that bring back memories of the event, negative cognitive symptoms and hyper arousal symptoms including difficulty sleeping and trouble concentrating [11]. It is common for such symptoms to occur in the first few weeks following exposure to a traumatic event, however, persistence of such symptoms for more than a month may mean that one has developed PTSD. Patients with a diagnosis of PTSD may also have some psychiatric comorbidities such as anxiety and depression, and substance abuse and addictions [14]. Also, an increased risk of suicide among those with PTSD has been found [46].

Various studies have shown that police officers are at a higher risk of developing PTSD with one study among police officers in the USA reporting a PTSD rate of 13% (Robinson, 1997). Another study among Dutch police officers reported a rate of 7% for those who met the full criteria for PTSD and 34% for those who met sub-threshold criteria for PTSD [6]. A retrospective study among riot police officers in Cape Town and another among black police officers in Soweto and Pretoria indicated that 36% of the riot police officers and 41% of black police officers respectively suffered from PTSD [8].

There is limited information on work related trauma and its mental health consequences including PTSD among police officers in sub-Saharan Africa including Uganda. The purpose of this study was to help address this gap by investigating work related trauma, PTSD and associated factors among police patrol officers working in the Kampala Metropolitan Police Region, Uganda.

## Method

### Study design, setting and population

The study was a cross sectional study of post-traumatic stress disorder conducted in Kampala Metropolitan Police North region (KMP) among field police patrol officers (FPPOs) serving in KMP North. KMP North is one of the 27 policing regions of the Uganda police force (UPF). The functions of the UPF involve among others; assisting victims of fatal accidents, handling cases of ritual murders, rape and defilement, dispersing unlawful crowds like riots and apprehending criminals, which can involve an exchange of gunfire [15], police, [9]. According to the Uganda Police Force statistical abstract of 2015, the lowest rank on the ladder is the probationary police constable. From there, the ranks move up to Police Constable referred to as junior officers in this study; the ranks that follow include Corporal, Sergeant, Assistant Inspector of Police (AIP), and Inspector of police (IP) which are all referred to as middle rank in this study. Above these ranks are the Assistant Superintendent of Police (ASP), Superintendent of Police (SP), Commissioner of Police (CP), Assistant Inspector General of Police (AIGP), Deputy Inspector General of Police (DIGP) and finally the Inspector General of Police (IGP) who are referred to as senior officers in this study. The Uganda Police Force has 44,897 officers serving country-wide, of which 7,700 (17.2%) are female and 37,197 (82.8%) are male, and 83 percent hold the rank of sergeant or below. KMP North is a political region encompassing two districts namely Kampala and Wakiso districts. Kampala district is the capital city of Uganda and is found in the central Region. The city was estimated to have a population of 1,680,800 people on 31st July 2019. Wakiso District also lies in the Central Region of Uganda and lies approximately 20 km, by road, northwest of Kampala. KMP North has 1762 police officers who serve in 6 divisions which include Old Kampala, and Wandegeya (together referred to as Wandegeya division in this study); Kawempe and Kasangati (together referred to as Kawempe division in this study); Wakiso and Kakiri (together referred to as Kakiri division in this study) [9].

This study was done among police officers who were carrying out duties including patrolling, arresting criminals, controlling riots, helping accident victims and handling dead bodies. These police officers are referred to as field police patrol officers (FPPO) in this study. All FPPOs serving in KMP North region who met the study criteria were considered in this study.

### Selection criteria

The study included all FPPOs serving in the KMP North region who gave consent to be part of the study and excluded all police officers who had served in the police force for less than one month.

### Sampling method

We used simple random sampling to select our study participants. After obtaining written permission from the Uganda police research department, we also obtained permissions from the various police commanders and head of stations to ensure cooperation with the study.We obtained a list of the field police patrol officers on duty that day from the officers in charge of stations (O.C). We selected every third officer on the list to be part of the study. The O.C. Station then helped us trace the selected study respondents to their assigned areas of work and we interviewed them from there.

### Study procedure

Data collection was done by the Principal investigator and two research assistants (RAs) who were both qualified psychiatric clinical officers and had prior experience in carrying out psychiatric research. As part of preparations prior to data collection, the RAs received training from the P.I on the data collection procedures and on how to administer the various study tools. We notified the O.C. Station a day before we visited a particular police station. The O.C. Station then informed FPPOs under his/her command about our impending visit. At each police station we selected potential study respondents by sampling from the list of FPPOs who had come to work. We then sorted out these potential study respondents from the areas where they had been deployed. We carried out interviews five days per week using the study tool. For every police officer we approached, we introduced ourselves and the study. All the police officers we approached were receptive and gave informed consent prior to administering the study tools. All officers who reported any symptom of a psychiatric disorder (e.g., PTSD, depression, alcohol use disorder, etc. were referred to Mulago mental health clinic for further assessment and management. Each filled in questionnaire was labeled with a unique study number and then locked away in a safe place by the Principal investigator pending data entry.

## Definitions and study measures

### Post-Traumatic Stress Disorder

PTSD was defined using the diagnostic criteria in the DSM-5 [1]. To meet criteria for PTSD, individuals need to have at least one exposure to a traumatic event (criterion A), at least one (of five) intrusion symptoms (criterion B), at least one (of two) avoidance symptoms (criterion C), at least two (of seven) negative cognitions and mood symptoms (criterion D), at least two (of six) reactivity and arousal symptoms (criterion E), duration of symptoms for at least one month (criterion F), and functional impairment (criterion G), which are not attributable to another condition (criterion H). Sub threshold PTSD was defined as the presence of at least one PTSD symptom but not meeting full criteria for PTSD diagnosis. Delayed onset of symptoms was defined as PTSD symptoms that occur 6 months after the traumatic experience.

The DSM-IV-TR specifically defines a trauma as direct personal experience of an event that involves actual or threatened death or serious injury, or other threat to one’s physical integrity; or witnessing an event that involves death, injury, or a threat to the physical integrity of another person; or learning about unexpected or violent death, serious harm, or threat of death or injury experienced by a family member or other close associate [2].

PTSD was assessed with the Clinician Administered PTSD Scale for DSM-5 (CAPS-5), past month version. The Clinician-Administered PTSD Scale (CAPS) is a structured diagnostic interview that assesses posttraumatic stress disorder (PTSD) diagnostic status and symptom severity. The CAPS is considered as the gold standard clinical interview to establish the diagnosis of PTSD [55] it measures DSM-5 PTSD symptoms, duration of symptoms, and global impairment and functioning related to symptoms. For each diagnostic criterion, interviewers rate on a scale from 0 (absent) to 4 (extreme/incapacitating) using information on both frequency and intensity of symptoms obtained during the interview. Items with a score of ≥ 2 and a functional impairment score of ≥ 1 are counted toward diagnosis. Although the CAPS has not been used in Uganda, it has been used as a gold standard measure of PTSD in research in Zimbabwe, which is a low and middle income country like Uganda [52].

### Socio demographic variables

Using the socio-demographic questionnaire, we collected information on sex, age, and tribe, highest level of education, marital status, and gross salary each month from the participants.

### Occupation related factors

The following police occupation related factors were assessed: Current police rank, duration in the police force, duration of service at KMP North, duration of service as a patrol police officer, and previous service in the army.

### Psychiatric comorbidity

In order to assess for psychiatric comorbidities, the study used the Mini International Neuropsychiatric Interview (M.I.N.I.). The comorbidities assessed include depression, generalized anxiety disorder, alcohol use disorder and suicidality. The M.I.N.I. is a diagnostic structured interview that was developed for DSM-5 psychiatric disorders [39]. The M.I.N.I. has been used quite extensively to assess for psychiatric disorders in research in Uganda [38].

### Data analysis

Data was entered and analyzed using Epi data and STATA version 14 with the help of a medical statistician. Frequencies and proportions were used to summarize categorical variables. Continuous variables were summarized using mean and standard deviations. To determine the prevalence of PTSD among field police patrol officers in KMP North, the number of police patrol officers with PTSD were obtained as a percentage (and its confidence interval) of the total number of study participants. To determine the factors associated with PTSD, we used simple logistic regression to obtain unadjusted odds ratios at bivariate analysis. Variables that showed associations with a cut off threshold *p*-value < 0.200, did not cause multi-collinearity and didn’t have sparse data in categories were considered at the multivariable analysis using stepwise elimination to obtain adjusted odds ratios and their confidence intervals. At multivariable analysis, a multivariable logistic regression model was used to obtain factors that were independently associated with PTSD at *p* < 0.050 and 95% confidence interval.

## Results

**Fig. 1:**
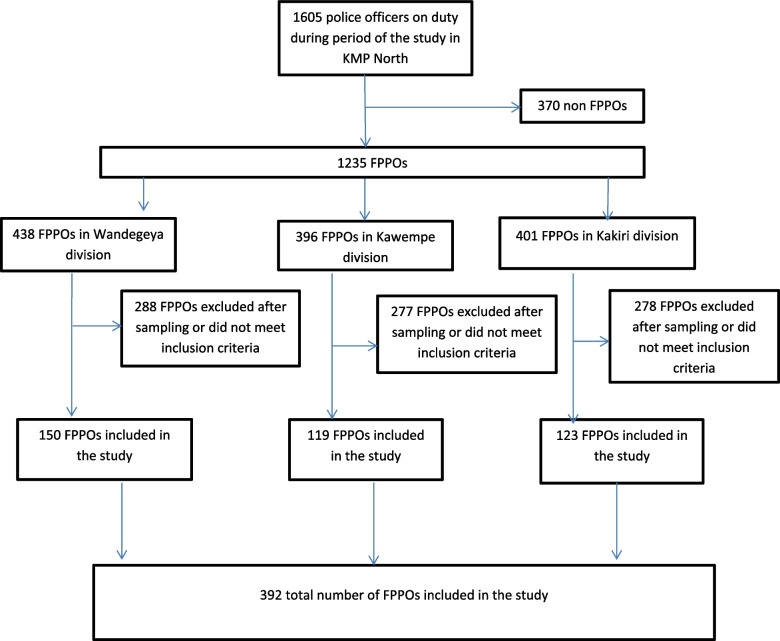
Flow chart for the recruitment process of the police officers

A total of 392 field police patrol officers (FPPO) were interviewed for this study from all the police divisions in Kampala Metropolitan police region North (KMP North). The Fig. 1 below is a Flow chart showing the recruitment process of the police officers.

Table 1 below shows the socio- demographic characteristics of the FPPOs.

**Table 1:** Socio- demographic characteristics of the population

Socio-demographic characteristic	*N* = 392 (100%)
**Division**	
Wandegeya	150 (38.3)
Kawempe	119 (30.4)
Kakiri	123 (31.4)
**Gender**	
Female	85 (21.7)
Male	307 (78.3)
Age; Mean (st.dev)	34.86 (7.88)
**Age**	
20 to 25	11 (2.8)
26 to 30	138 (35.2)
31 to 40	164 (41.8)
> 40	79 (20.2)
**Marital status**	
Single	82 (20.9)
Married	305 (77.8)
Separated	5 (1.3)
**Religion**	
Christian	321 (82.0)
Muslim	38 (9.7)
Others	33 (8.3)
**Education Level**	
11 years of school	99 (25.3)
13 years of school	169 (43.1)
More than 13 years of school	124 (31.6)
**Region**	
Northern	103 (26.4)
Eastern	135 (34.4)
Central	40 (10.2)
Western	88 (22.4)
Others	26 (6.6)

### Work related characteristics of the police officers

Table 2 below shows the work related characteristics of the FPPOs enrolled into this study.

**Table 2:** Work related characteristics of the police officers

Service characteristics	*N* = 392 (100%)
**Police rank**	
Junior officer	304 (77.6)
Middle rank	64 (16.3)
Senior officer	24 (6.1)
**Duration in Police**	
Below 1 year	1 (0.3)
1 to 5 years	171 (43.6)
> 5 years	220 (56.1)
**Duration in KMP North**	
Below 1 year	14 (3.6)
1 to 5 years	218 (55.6)
> 5 years	160 (40.8)
**Duration as Patrol officer**	
Below 1 year	27 (6.9)
1 to 5 years	209 (53.3)
> 5 years	156 (39.8)
**Monthly income (USD)**	
< 135 USD	311 (79.3)
135 to 270 USD	79 (20.2)
> 270 USD	2 (0.5)
**Previous service in the Army**	
No	376 (95.9)
Yes	16 (4.1)

### PTSD and other Psychiatric Comorbidities

Table 3 below, of the 392 interviewed respondents 29 (7.4%) met full criteria for PTSD; 245 (62.5%) had sub-threshold PTSD symptomatology while 118 (30.1%) had no PTSD symptoms. Out of the 245 respondents who had sub-threshold PTSD, 66 (27%) of them reported being impaired or distressed in at least one of the spheres of work, social or any other important area of functioning. Out of the 274 police officers who had at least one symptom of PTSD, 25 (9.1%) reported delayed onset of symptoms.

**Table 3:** Prevalence of PTSD

PTSD symptom characteristic	Frequency (*N* = 392)	Percentage
No PTSD symptoms	118	30.1
Symptomatic but did not meet PTSD criteria	245	62.5
PTSD criteria met	**29**	**7.4**
Delayed onset of symptoms (*n* = 274)	25	9.1

**Fig. 2:**
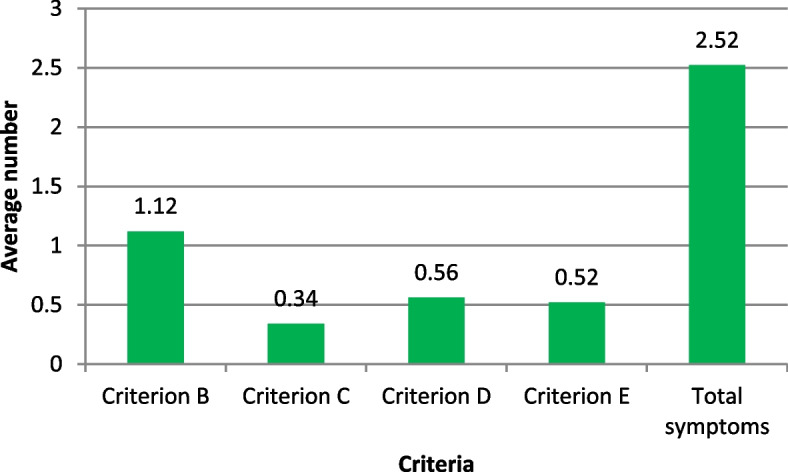
Graph showing the average numbers of Criteria B, C, D, E and total number of symptoms in symptomatic police officers, KEY: Criterion B- *Intrusive symptoms,* Criterion C- *Avoidance symptoms*, Criterion D- *Cognitive symptoms*, Criterion E- *Hyper arousal symptoms*

On the pattern of PTSD symptomatology experienced by the respondents, the average number of symptoms experienced by a police officer in this study was 2.5 with a standard deviation of 2.9. Most of the PTSD symptoms experienced were of Criterion B (see Fig. 2 below).

### Psychiatric comorbidities

Table 4 below shows the psychiatric disorders that were comorbid with PTSD.

**Table 4:** PTSD psychiatric comorbidities

**Psychiatric comorbidities**	**Frequency (*N* = 392**)	**Percentage (100%)**
No psychiatric comorbidity	159	40.6
CMD only	7	1.8
PMD only	9	2.3
AUD only	52	13.3
GAD only	18	4.6
Presence of Dissociative symptoms	29	7.4
CMD and PMD	8	2.0
CMD and AUD	4	1.0
CMD and GAD	36	9.2
PMD and Suicidality	6	1.5
PMD and GAD	10	2.6
AUD + GAD	18	4.6
CMD + PMD + AUD	9	2.3
CMD + PMD + GAD	6	1.5
CMD + Suicidality + GAD	3	0.8
CMD + AUD + GAD	12	3.1
PMD + Suicidality + GAD	3	0.8
PMD + AUD + GAD	3	0.8

*CMD* Current Major Depression, *PMD* Past Major Depression, *AUD* Alcohol use Disorder, *GAD* Generalized Anxiety Disorder

### Nature of the worst traumatic events experienced by the respondents

For one to develop symptoms of PTSD, they must have experienced a traumatic event. Table 5 below, describes the nature of the worst traumatic events experienced by the police officers. Seeing dead bodies (16.8%) was the most traumatic event reported followed by witnessing accidents (14.3%). The traumatic events that were grouped as ‘others’ include being taken hostage, involved in ambush, being attacked by an animal, and being deployed in conflict affected Somalia.

**Table 5:** Nature of the worst traumatic events experienced by respondents

Traumatic event	Frequency (*N* = 392)	Percentage (100%)
Seeing dead bodies	66	16.8
Witnessing accidents	56	14.3
Officer being killed on duty	51	13.0
Being injured while on patrol	46	11.7
Being involved in shoot outs with criminals	44	11.2
Seeing mob justice	24	6.1
Carrying dead bodies	22	5.6
Seeing someone dying	18	4.6
Being involved in stopping riots	12	3.1
Handling rape victims	7	1.8
Could not discuss the event	4	1.0
Others	42	10.7

**Fig. 3:**
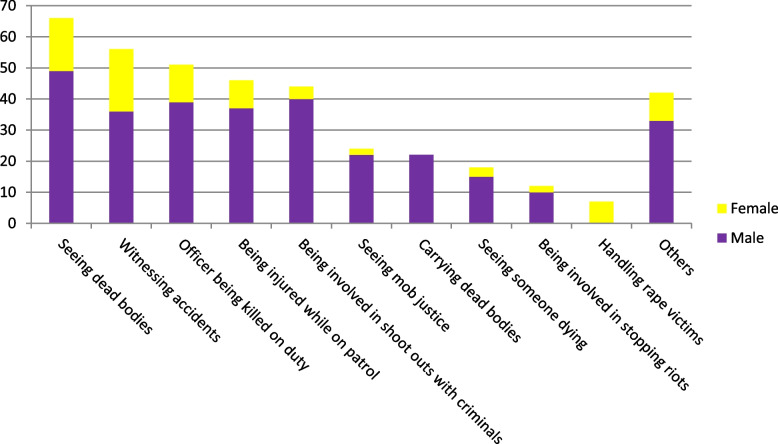
Nature of all traumatic events experienced by the police officers by gender

Figure 3 below shows the frequency of the reported traumatic events by gender.

### Association between PTSD and other psychiatric comorbidities among the respondents

Table 6 below shows the association between PTSD and other psychiatric comorbidities of the respondents. The psychiatric comorbidities that were significantly associated with PTSD were: presence of dissociation symptoms (*p* < 0.001), current major depression (*p* < 0.001), past major depression (*p* = 0.006), and alcohol use disorder (*p* = 0.001).

**Table 6:** Association between PTSD and psychiatric comorbidities among respondents

Symptom characteristic	PTSD [*N* = 29(%)]	No PTSD [*N* = 363 (%)]	*p*
Presence of Dissociation symptoms	10 (34.5)	19 (5.2)	** < .001**
Current major depression	9 (31.0)	27 (7.4)	** < .001**
Past major depression	6 (20.7)	24 (6.6)	**.006**
Suicidality	2 (6.9)	6 (1.7)	.112
Alcohol use disorder	12 (41.4)	59 (16.3)	**.001**
Generalized anxiety disorder	6 (20.7)	53 (14.6)	.378

### Factors independently associated with PTSD at multivariate analyses

Prior to multivariate analysis, individual associations between independent variables and PTSD were assessed using individual logistic regressions. Variables that showed associations with a cut off threshold *p*-value < 0.200, did not cause multi-collinearity and didn’t have sparse data in categories were considered at the multivariable analysis. At multivariable analysis, a multivariable logistic regression model was used to obtain factors that were independently associated with PTSD at p < 0.050 and 95% confidence interval.

Table 7, factors that were independently significantly associated with PTSD at multivariate analyses were all related to the presence of psychiatric comorbidities, namely: presence of dissociation symptoms [aOR = 6.7; 95% CI (2.0–22.2), *p* = 0.002]; having a current major depressive episode [aOR = 4.7; 95% CI (1.5- 14.8), *p* = 0.009]; and having an alcohol use disorder [aOR = 5.1; 95% CI (2.0–13.0), *p* = 0.001].

**Table 7:** Multivariate logistic regression showing factors associated with PTSD

Characteristics	UNADJUSTED OR (95% CI)	*P*	ADJUSTED OR (95% CI)	*p*
**Age**				
20–25	**1.0**		**1.0**	
26–30	0.8 (0.1–6.7)	.820	2.2 (0.1–34.5)	.570
31–40	0.9 (0.1–7.3)	.890	1.5 (0.1–22.8)	.770
> 40	0.7 (0.1–6.4)	.730	1.1 (0.1–19.2)	.930
**Gender**				
Female	1.00		1.0	
Male	1.07 (0.4–2.7)	.900	1.4 (0.4–4.7)	.580
**Religion**				
Christian (*n* = 321)	1.0		1.00	
Muslim (*n* = 38)	2.4 (0.92—6.41)	.073	2.38 (0.72—7.89)	0.160
Others (*n* = 33)	-		1.00	
**Region**				
Northern (*n* = 103)	1.0		1.0	
Eastern (*n* = 135)	2.0 (0.8–5.4)	.160	2.2 (0.7–7.1)	.190
Central (*n* = 40)	0.9 (0.2–4.4)	.850	1.5 (0.3–9.7)	.640
Western (*n* = 88)	0.8 (0.2–2.8)	.690	0.6 (0.1–2.9)	.510
Others (*n* = 26)	1.4 (0.3–7.1)	.730	0.5 (0.0–6.4)	.560
**Army service**				
No (*n* = 376)	1.0		1.00	
Yes (*n* = 16)	3.1 (0.8–11.6)	.090	3.6 (0.7–19.0)	.130
**Presence of Dissociation symptoms**				
No (*n* = 363)	1.0		1.0	
Yes (*n* = 29)	7.2 (2.8—18.5)	< .001	6.7 (2.0–22.2)	**.002**
**Current major depressive episode**				
No (*n* = 356)	1.0		1.0	
Yes (*n* = 36)	5.6 (2.3–13.5)	< .001	4.7 (1.5–14.8)	**.009**
**Past major depressive episode**				
No (*n* = 362)	1.0		1.0	
Yes (*n* = 30)	3.7 (1.4–9.9)	.010	2.0 (0.5–8.0)	.330
**Suicidality**				
No (*n* = 384)	1.0		1.0	
Yes (*n* = 8)	4.4 (0.9- 22.9)	.080	2.3 (0.3–18.2)	.420
**Alcohol use disorder**				
No (*n* = 321)	1.0		1.00	
Yes (*n* = 71)	3.6 (1.7–8.0)	.001	5.1(2.0–13.0)	**.001**

**Note: *OR* Odds ratio, *CI* Confidence interval**

## Discussion

The study investigated the prevalence of PTSD among police officers whose main work is to carry out patrols in the various parts of Kampala Metropolitan region north. The study also investigated the factors that are associated with PTSD in this group of police officers. We interviewed a total of 392 field police patrol officers.

### Socio-demographic characteristics of the police officers

The average age of the respondents in this study was 34.8 years with the most represented age group being those aged between 31 to 40 years. This means that most police officers in this study were young adults with similar findings reported by Steyn and colleagues (2013) in South Africa who reported that the average age of the respondents in his study was 40 years [44]. Our findings are also similar to a study done among Brazilian police officers, where the mean age of the officers was 34.8 years old [24]. However, police officers in a study done by Kristinsdóttir et al., K. (2018) among police officers in Iceland were much older than those found in our study with most of them being in the age of 40 to 49 years [20].

There were more male respondents (78.3%) than females (21.7%) in this study. Similar gender ratios have been reported elsewhere in the USA, Iceland and in South Africa [19, 20, 44]. This is probably because police work is traditionally considered a male job.

Most of the respondents in this study were married (77.8%) which is similar to a study by Tay and colleagues (2017) in Timor- Leste, a country in Asia, where the majority of respondents were married [47].

Most of the respondents in this study had attained at least 13 years of school education. This is similar to the findings in a study by Steyn and colleagues [44] in South Africa. This is probably because recruitment into the police officer usually requires a minimum number of years of formal education.

### Work related characteristics of the respondents

Most of the respondents in this study were junior officers (77.6%). This is probably because the majority of the persons deployed in the field tend to be from the lower ranks. Hartley and colleagues (2013) in a study of PTSD in the USA also reported that the majority of the police officers in their study were junior officers [12].

The majority of police officers in this study had served for more than five years in the police force. Similar findings were reported in a study from the USA by Violanti and colleagues (2018) where the police officers had served in the police force for more than 10 years [54].

The police service, like many other government departments provide employment with security. Despite job security, the pay in the police force was mostly poor with the majority of police officers in this study earning less than 135 US Dollars per month.

### Traumatic experiences among police officers

The most commonly reported traumatic events in our study were seeing dead bodies, witnessing accidents, officer being killed on duty and being injured while on patrol. These findings are similar to those reported in other studies where the most reported traumatic events were: witnessing gunshots that put one’s own life or a coworker’s life in danger; and witnessing child abuse, death, including homicide victims, or victims of serious traffic accidents [10, 28, 53]. This study found that although majority of the police officers interviewed narrated at least one traumatic event, four police officers could not discuss the details of the traumatic event they may have experienced. It is very unlikely that these police officers had not experienced a traumatic event during their day to day work [17]. It was probably that they had been so traumatized by the event that they did not want to relive the traumatic experience again. Another possibility could be that the traumatic event involved a sensitive subject such as shooting at someone and they felt they could not discuss this with a civilian researcher as it was not socially acceptable [34].

### Prevalence of PTSD among police patrol officers

The study found a prevalence of PTSD to be 7.4% among field police patrol officers serving in Kampala Metropolitan region north. The study also found that 62.5% of field police patrol officers had sub-threshold symptoms of PTSD. The prevalence of PTSD in this study was similar to that reported in a study undertaken among Dutch police officers where a rate of PTSD of 7% and a rate of sub-threshold PTSD of 34% were reported [6]. Other studies undertaken in Brazil, USA, and Canada reported rates of PTSD of between 6 and 9%, in agreement with findings from this study [23, 25, 26] respectively). However, the results from this study were much lower than those reported from another African country. Edwards [8] in a study undertaken among riot police officers in Cape Town, South Africa reported a rate of PTSD of 36%. The same author reported among black police officers in Soweto, Johannesburg and Pretoria a prevalence of PTSD of 41% [8]. Strommes, JM [45] in an earlier study in Kwazulu Natal, South Africa reported among police officers a prevalence of PTSD of between 12 and 35% [45]. This may reflect the underlying high levels of community violence in South Africa compared to Uganda, a legacy that South Africa draws from its Apartheid history [42].

### Factors associated with PTSD

The factors found to be associated with PTSD in this study were all related to the presence of psychiatric comorbidities, namely presence of dissociation symptoms, having a current major depressive episode and having concurrent alcohol use disorder.

Our findings are similar to those reported in both the National Comorbidity Study in the United States by Kessler and colleagues [18] and in the study by Breslau and colleagues [4] undertaken among young adults in Detroit (USA). In both studies, it was reported that individuals with PTSD also met criteria for another psychiatric disorder [4, 18]. In the study by Breslau and colleagues [4] the psychiatric disorders that were most comorbid with PTSD were substance abuse or dependence (43%), major depression (37%) and agoraphobia (22%) [4].

In more recent study by Pietrzak and colleagues [35] undertaken among police officers who were involved in the World Trade Center rescue, comorbidity between PTSD with depression, panic disorder, and alcohol use problems was common [35]. These findings are similar to our study findings where PTSD was significantly associated with current major depression and alcohol use disorder. Our study also found a significant association between PTSD and dissociation symptoms. Hodgin and colleagues (2001) among police officers in Australia found that one of the risk factors for development of PTSD was having a dissociation trait [13]. Meta-analyses conducted by Ozer et al. [32, 3] and Lensvelt-Mulders et al. [21], confirm that peritraumatic dissociation is one of the risk factors in the development of PTSD [3, 21, 32].

In this study age, gender, marital status and work related factors were not significantly associated with PTSD among police officers. Similar findings to these reported in this study have been reported by other authors: on age [12, 41], on gender [5, 22, 36, 37, 48],on marital status [45]; and work related characteristics [12, 26, 29].

### Strengths and limitations of the study

This study is among the few studies that have looked at the prevalence and factors associated with PTSD among police officers in a low and middle income country. The study also contributes to the literature from low and middle income countries on the types of traumatic experiences those police officers in these settings experience. As a strength of this study, we used a DSM-5 referenced PTSD assessment tool, the Clinician Administered PTSD scale (CAPS) which is regarded as the gold standard for PTSD diagnosis with good internal consistency and reliability [55]. Additionally, our study had an adequate sample size which was taken from multiple sites in a reasonably large region. We could therefore generalize our findings to all field police officers in Uganda.

On study limitations, this being a cross-sectional study we could not determine the direction of causality between PTSD and the associated factors. Secondly, some of the variables we used suffered from recall bias as relied on memory of the study participants.

Thirdly, we had challenges accessing all the police officers in the division; we could only access those police officers who were on duty. Lastly, due to the fact that PTSD symptoms can be distressing, we could not determine if all those interviewed were honest with their responses when interviewed face to face.

## Conclusions

Serving field police officers in Kampala Metropolitan had a considerable burden of PTSD with a tendency to co-occur with the psychiatric disorders/symptoms of major depression, alcohol use disorder and dissociation symptoms. Therefore there is need to establish a service to screen for and treat PTSD among serving police officers in Uganda as part of a multi-disorder mental health treatment program in the Police force. There is need for more research to gain a deeper understanding of the mental health problems of the police officers in low and middle income settings such as those in Uganda.

## Data Availability

The dataset(s) supporting the conclusions of this article has been provided in the manuscript text and tables.

## Abbreviations

CAPS: The Clinician-Administered PTSD Scale
DPC: Division police commander
DSM 5: Diagnostic and Statistical Manual of Mental Disorders, Fifth Edition
DSM IV TR: Diagnostic and Statistical Manual of Mental Disorders-Fourth Edition (Text Revision)
FPPO: Field Police Patrol Officers
KMP: Kampala Metropolitan Police
M.I.N.I: Mini International Neuropsychiatric Interview
O.C: Officer in charge
PTSD: Post traumatic Stress Disorder
UPF: Uganda Police Force
